# Role of the Pharmacist in Supporting the Use of Connected Health Devices: Example of Connected Watches

**DOI:** 10.3390/pharmacy14010028

**Published:** 2026-02-03

**Authors:** Cordélia Salomez-Ihl, Léa Liaigre, Wiceme Dala, Ambre Davat, Maud Barbado, Sébastien Chanoine, Philippe Py, Delphine Schmitt, Pascal Defaye, Pierrick Bedouch

**Affiliations:** 1 University of Grenoble Alpes, CNRS, UMR5525, Grenoble INP, TIMC, Pharmacy Department, CHU Grenoble Alpes, 38000 Grenoble, France; cihl@chu-grenoble.fr (C.S.-I.); lliaigre@chu-grenoble.fr (L.L.); wiceme99@gmail.com (W.D.); schanoine@chu-grenoble.fr (S.C.); 2 University of Grenoble Alpes, Pharmacy Department, CHU Grenoble Alpes, 38000 Grenoble, France; ppy@chu-grenoble.fr (P.P.); dschmitt@chu-grenoble.fr (D.S.); 3 University of Grenoble Alpes, GRESSEC Laboratory, 38000 Grenoble, France; 4 University Grenoble Alpes INSERM 11 CIC1406, Clinical Investigation Center-Technological, Innovation,Public Health Department, CHU Grenoble Alpes, 38000 Grenoble, France; mbarbado@chu-grenoble.fr; 5 University of Grenoble Alpes, CNRS, UMR5525, Grenoble INP, TIMC, Cardiology Department, CHU Grenoble Alpes, 38000 Grenoble, France; pdefaye@chu-grenoble.fr

**Keywords:** connected medical device, clinical pharmacist, electrophysiology, therapeutic education

## Abstract

The use of Connected Medical Devices (CMDs) is growing significantly throughout the world. Although they are not dispensed in pharmacies and are not part of the pharmacy-only drug dispensing system, clinical pharmacists must be able to support patients in the use of these new technologies, which are central to their care. The aim of this study is to identify the role of the community pharmacist in supporting patients who use CMDs, using the case of connected watches in electrophysiology. Semi-structured interviews were conducted between 15 February and 20 April 2024 by a pharmacy student. The questionnaires were drafted in collaboration with a pharmacist, a cardiac electrophysiologist, a methodologist specializing in the evaluation of medical devices, and an ethical philosopher specializing in the support and acceptability of new technologies. The aim of these questionnaires was to study the use of connected watches and support for patients who own them. A total of 4 cardiac electrophysiologists and 10 cardiac electrophysiology patients were interviewed, and then 6 pharmacists were also questioned about the roles identified by physicians and patients. This study identified a major need on the part of specialist physicians for clinical pharmacist support in helping patients use connected watches. Patients expressed a high level of confidence in their pharmacists to support them, and in the motivation of pharmacists’ ability to take up these challenges. A number of challenges remain, such as the effective integration of this support into pharmacy practice, remuneration, and the organization of collaboration between clinical pharmacists and hospital electrophysiologists.

## 1. Introduction

E-health is defined by the World Health Organization as the use of Information and Communication Technologies (ICTs) for health [1]. This practice is developing significantly, as is the use of Connected Health Objects (CHOs) [2] and, more specifically, Connected Medical Devices (CMDs) [3,4,5]. Although CMDs are fully fledged healthcare products, they are not under the pharmacy-only drug dispensing system. A CMD is a CHO for which there is a medical claim. This claim may be therapeutic or diagnostic, for example. Once a CHO becomes a CMD, it must comply with the regulations in force concerning medical device (MD) status. This usage is even more prominent in a healthcare system faced with an aging population [6,7] and an increasing prevalence of chronic diseases [8,9]. Indeed, CMDs provide healthcare professionals and patients with chronic conditions the ability to remotely monitor and interpret medical data [10,11,12].

CMDs are used in numerous therapeutic areas. In the context of cardiovascular diseases, the most commonly used CMDs are aimed at diagnosing and monitoring conditions, such as connected blood pressure monitors for hypertension and smartwatches for rhythm disorders. Some CMDs are also implantable, particularly for treatment, monitoring, and remote surveillance, such as pacemakers and automatic defibrillators [13,14]. The proper use of these CMDs relies on patient support by healthcare professionals. Community pharmacists already play a key role in supporting patients with chronic diseases, notably through therapeutic education [15,16,17]. They hold an essential role in the healthcare system as they are in close proximity to patients and serve as first-line providers [18]. Therefore, their involvement in supporting patients using CMDs is crucial [19].

To ensure effective patient follow-up, community pharmacists face several challenges. Healthcare professionals need to be trained in the use of CMDs and in supporting patients who own them. Furthermore, most CMDs are not distributed in community pharmacies, and the support provided to patients using CMDs is currently not recognized as a pharmacist’s role, which explains the lack of associated remuneration. Lastly, not all CHOs are qualified as CMDs. Only objects with a medical claim can bear this designation, which comes with an EC (European Conformity) marking [20]. It is therefore important to precisely determine whether patients are using CMDs or CHOs in a landscape where an increasing number of technological innovations with various statuses coexist.

Connected watches represent a major advancement among CMDs in cardiac electrophysiology [21]. Equipped with sensors such as photoplethysmographs and sometimes integrated electrocardiograms (ECGs), they enable continuous and non-invasive monitoring of heart rate, rhythm, and other physiological parameters. These devices play a key role in the early detection of arrhythmias, particularly atrial fibrillation, thanks to advanced analysis algorithms validated by clinical studies, even in neonates [22]. In recent years, their use has revolutionized the diagnosis of intermittent atrial fibrillation, enabling the detection of events that would not previously have been seen with the usual diagnostic methods, in particular, the Holter ECG. Complementing traditional medical monitoring, smartwatches provide patients with greater autonomy and increased awareness of their health status while generating data usable by clinicians for personalized longitudinal follow-up. Their integration into rhythmological care opens promising perspectives but also raises challenges related to interoperability, data reliability, and security management.

The objective of this study was to identify the roles of community pharmacists in supporting patients using CMDs, using the example of connected watch usage in cardiac electrophysiology.

## 2. Materials and Methods

**Figure 1:**
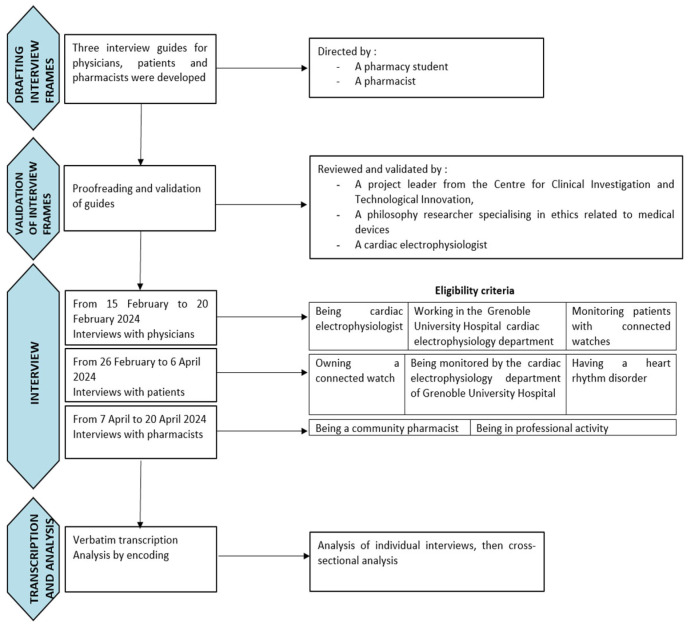
Flowchart of the study.

Semi-structured interviews were conducted with three study groups: patients, rhythmologists, and community pharmacists. Patients and physicians were interviewed between 15 February and 6 April 2024. Pharmacists were interviewed from 7 April to 20 April 2024. Figure 1 illustrates the study organization.

Initially, three interview frameworks were developed by a pharmacist and a pharmacy student (Appendixes A1 to A3). Each interview guide consisted of open-ended questions and follow-up prompts, arranged in a predefined sequence. The guides followed the same structure and were complementary. They were divided into two parts: the first aimed to collect data on the participant’s profile, while the second was organized around three themes: the device, patient support, and the role of the community pharmacist. Each section included sub-themes with an open-ended question and an associated follow-up prompt. The final section was dedicated to evaluating the interview itself, using a scale from 1 to 10 to assess factors such as the interview duration, methodology, topics covered, and the relevance of the questions.

The interview frameworks were reviewed and validated by a project coordinator from the Clinical Investigation and Technological Innovation Center, a philosophy researcher specializing in ethics related to medical devices, and a rhythmologist from the institution. The institution’s Clinical Research and Innovation Department was informed of the project and certified that this study did not require approval from an ethics committee. The questionnaires were tested and validated during an initial interview with a physician, a patient, and a pharmacist.

In line with French legislation for prospective studies using clinical data, participants were informed individually that their data could be used for research purposes. Those who objected to the use of their data were excluded from the study. Oral consent was obtained from patients, physicians, and pharmacists regarding the following points: the academic use of the data and the recording of conversations.

Participants were subsequently recruited. Interviews were offered to all rhythmologists in the team at the Grenoble Alpes University Hospital (GAUH). Patients were selected from the institution’s active cardiac electrophysiology patient database, specifically those monitored with connected watches, based on their availability and recent interactions with the medical team. The pharmacists were recruited following a random draw from an official list of retail pharmacies available at the Faculty of Pharmacy. Their views on the subject under study were not known prior to meeting them. No member of the team was able to influence the views of the pharmacists surveyed. Eligibility criteria are detailed in Figure 1.

All participants were contacted by email and interviews were conducted face to face. Interviews with physicians were conducted first, from 15 to 20 February 2024, followed by patients from 26 February to 6 April 2024, and finally pharmacists from 7 to 20 April 2024. Interviews were conducted by a community pharmacy student with prior experience in conducting interviews during shared medication review processes in community pharmacies. The interviews were supervised by a pharmacist, with the guide serving as a reference. Before each interview, participants were informed about anonymity, the time required, and complete transparency regarding the study’s objectives and the use of collected data.

Each interview was individually and fully transcribed and analyzed in an initial phase. Data were analyzed through coding, which involved identifying relevant text segments manually and assigning them appropriate codes. The codes were then grouped by theme to organize and facilitate interpretation. In the second phase, a cross-thematic analysis was performed, using tables to categorize themes and codes from interviews with physicians, patients, and pharmacists. The pharmacy student proposed a grouping, and a senior review validated the proposed thematic groupings (Appendixes B1 to B3).

## 3. Results

Among the six rhythmologists contacted, four responded and participated in the study. All contacted patients (*n* = 10) and pharmacists (*n* = 6) participated. The characteristics of the interviewed population are summarized in Table 1.

The interviews conducted with the physicians highlighted the following themes: the device, added value, funding, support for physicians, usage by patients, and the role of the community pharmacist.

Table 2 illustrates the key results obtained from the interviews with the physicians.

All of the physicians interviewed used smartwatches to diagnose rhythm disorders in their daily practice. According to them, the watches provided a new, effective diagnostic tool by allowing recordings during symptoms for patients who had never recorded abnormalities with other diagnostic methods, such as Holter monitors, which could be less convenient and more restrictive.

For 75% of physicians, smartwatches were also useful for monitoring rhythm changes after surgery or medication adjustments (Table 2). Physicians recommended watches compatible with patients’ phones, with Apple Watch^®^, Withings^®^, and Samsung Ultra^®^ being common choices.

One physician opposed hospital investment in such devices, citing funding and management constraints. Patients were encouraged to record symptoms at home and share data with their rhythmologists. While 75% (*n* = 3) reported equal patient contact regardless of smartwatch use, one physician noted more frequent contact from smartwatch users.

One physician mentioned being more frequently contacted by patients who used watches.

Regarding the role of the community pharmacist, collaboration with community pharmacists was seen as beneficial by half of the physicians, especially for patients needing technical support. Pharmacists could assist with device usage and recordings, with secure messaging preferred for communication. However, one physician highlighted limitations, including the lack of financial incentives for pharmacists, as connected watches are not sold in pharmacies or covered by billable support activities.

Interviews with patients revealed themes including watch types, benefits, costs, physician support, usage, education, psychological effects, and the role of pharmacists. Patients used Withings (Withings, Issy-les-Moulineaux, France), Apple Watch^®^ (Apple Inc., Cupertino, CA, USA), Fitbit^®^ (Google LLC, San Francisco, CA, USA), and Samsung Ultra^®^ (Samsung Electronics Co., Ltd., Gyeonggi-do, Republic of Korea), all featuring MD-certified software.

A total of 80% of patients (*n* = 8) reported receiving a diagnosis via their watch, with three noting difficulty in obtaining a diagnosis previously. Watches were also used for monitoring, with 40% (*n* = 4) sending ECG traces to physicians only for severe symptoms. Daily or weekly use depended on patient stability after procedures like atrial fibrillation ablation.

A total of 40% (*n* = 4) found the watches expensive, but 80% (*n* = 8) valued their benefits. Choices were influenced by personal research, relatives, or physician recommendations. Most patients felt autonomous, though 60% (*n* = 6) initially needed help from relatives. Two-thirds of patients (*n* = 6) found the watches easy to use after the initial period.

Psychologically, all patients found the watches reassuring, though 30% (*n* = 3) felt pressure from over-monitoring. A total of 90% (*n* = 9) trusted their pharmacist, with 30% (*n* = 3) discussing their watches with them. All patients believed pharmacists could help with usage issues, with 50% suggesting pharmacists offer usage support and 20% expecting training in connected watches and ECGs (Table 3).

Patients appreciated pharmacist support but noted limitations. One was wary of pharmacists interpreting rare conditions, and another highlighted the lack of financial incentives for pharmacist involvement.

Pharmacists reported encountering blood glucose meters, blood pressure monitors (33%, *n* = 2), and transcutaneous neurostimulation devices. In cardiology, connected blood pressure monitors were the most mentioned CMD, while smartwatches, oximeters, and connected rings were each noted once.

All pharmacists agreed that CMDs enabled regular monitoring, improved treatment adherence, and supported patient autonomy. Examples included blood pressure self-monitoring for antihypertensive adjustments, real-time glucose monitoring for pregnant women, and glucose monitoring for chronic disease management. One pharmacist had no direct experience with CMD applications (Table 4).

Half of the pharmacists noted risks of over-monitoring, while 33% (*n* = 2) mentioned potential excessive autonomy, reducing professional consultations. Another third highlighted issues with devices purchased outside medical circuits, including insufficient information provided at purchase.

All pharmacists saw their role as reassuring and supporting patients with CMD use, advising on device operation and data response. Half suggested implementing consultations or appointments in pharmacies, though 33% found this challenging without compensation: “The product would need to be sold in pharmacies, or there should be payment to motivate us.”

For collaboration, 80% (*n* = 5) preferred secure messaging with physicians, and a third supported an emergency phone line.

Among the obstacles to implementing this support, a lack of knowledge was consistently mentioned, while the lack of time was highlighted in four out of six interviews.

Regarding the solutions discussed, the main lever mentioned in all interviews was training, whether through continuing professional development, university programs, or laboratory training.

## 4. Discussion

To our knowledge, this is among the first qualitative studies to explore the role of community pharmacists in supporting CMDs in the field of cardiac electrophysiology. It is also the first to gather the opinions of physicians, patient users, and community pharmacists on this topic concerning a specific type of connected device. Finally, it is the first to explore the role of the pharmacist in providing therapeutic support for CMDs.

The study reveals a generally positive opinion from physicians and patients regarding the use of connected watches in cardiac electrophysiology. All physicians confirmed that they used connected watches both for diagnosis and patient follow-up. All the connected watch models used by physicians and patients in this study have an EC marking for their rhythm disorder detection software. Thus, the software of the watches used in this study are CMDs and not CHOs. Since these devices have medical purposes, this confirms their legitimacy for diagnosis and patient follow-up.

These devices are now central to the therapeutic management of patients with rhythm disorders. While the patients and physicians interviewed emphasized the ease of use of connected watches, they also confirmed that some patients, particularly the elderly, could experience difficulties using these technologically advanced devices. Indeed, Eurostat’s metadata reveals that Internet use decreases with age in most European countries. In 2024, only 61% of people aged 65–74 used the Internet regularly, compared with over 9% in younger age groups [23]. The physicians interviewed indicated that they tended to offer these watches to patients they believed could easily use the technology. To expand the use of connected watches to a larger number of patients, additional support beyond the physicians would be necessary, according to them. In this regard, the community pharmacist serves as a local support resource. Indeed, the results show that both patients and physicians trust community pharmacists to provide support for using these devices, provided they are better trained. Community pharmacists play a vital role as accessible public health resources in local communities. Several studies have investigated the expanding role of pharmacists and demonstrated their contribution to patient acceptability of healthcare interventions such as vaccination. Their clinical expertise and role in medication management make them key players in the successful adoption of AI for the benefit of patients in collaboration with other healthcare professionals [24,25].

However, training needs were highlighted by patients, physicians, and pharmacists themselves. Currently, the initial training for pharmacists is not focused on medical devices, especially connected ones. Increasing the training offer would help pharmacists improve their knowledge. For future generations of pharmacists, incorporating specialized modules on medical devices (CMDs) in their university curriculum is crucial. For currently practicing pharmacists, continuing education related to CPDs is desirable.

Other challenges were also raised by pharmacists, such as lack of time, organization, and the absence of specific compensation. Regarding the integration of this new role into pharmacy practice, implementing support programs similar to those already existing, such as consultations for pregnant women or patients on anticoagulants, could serve as a model [26]. To address the compensation issue, implementing fee-for-service payment, as is already the case for consultations with pregnant women, would recognize and value pharmacists’ additional work [27].

Finally, to successfully carry out this role, close collaboration between pharmacists and rhythmologists is essential. This collaboration must be well structured to be effective. Secure messaging, already in place, seems to be the preferred communication method for this collaboration [28]. Indeed, collaboration between pharmacists and physicians, and coordination between the city and hospital is crucial in managing patients across all medical fields.

National-level changes must therefore be implemented to address training and compensation issues. This is particularly important given that this survey reveals significant motivation among community pharmacists to take on this new role, as well as a need from physician and trust from patients.

The topic of supporting CMDs and connected health remains underdocumented. A study on the perception of connected health in France, with 1000 participants, was conducted in April 2024. Among the concerns related to the increasing use of connected health, 23% of patients expressed that tracking their health daily was anxiety-inducing, a sentiment also shared by three patients in this study [29]. Regarding community pharmacists, a study conducted with 200 pharmacists in France in 2020 explored their perception of health apps. When asked “Would you be willing to use this data in monitoring your patients?”, 46.4% of pharmacists answered positively, with 50% believing it was an effective way to improve adherence. This low proportion can be explained by the lack of information available to community pharmacists on this topic and the recent evolution of their roles [30]. Indeed, since 2020, community pharmacists have officially been able to engage in new roles such as vaccination, chronic disease management, teleconsultation, and telemedicine, including transmitting prescriptions through communication technologies.

The main limitations of this study are its monocentric nature for physicians and patients and the small number of participants. The low number of participants recruited, the lack of demographic data collection, and the fact that the patients interviewed were already ‘chronic’ users of smartwatches limit the external validity of the study. However, the diversity of the participants’ profiles contributes to the robustness of the study’s results. The patients interviewed were selected from those already using connected watches, which introduces a selection bias. It would have been interesting to speak with patients for whom this technology was not suitable. According to the clinical research unit’s opinion, patients’ ages could not be collected, due to confidentiality concerns. This parameter would have been useful, particularly to assess whether usability difficulties or differing perceptions were age-related.

The semi-structured interview framework ensured the consistency and quality of the data collected. It also allowed key themes to be addressed systematically, minimizing the roles or reformulations that could affect data comparability. The diversity of profiles that contributed to the development of the questionnaire was a definite asset in choosing and formulating the questions.

Future work should focus on implementing solutions to the various challenges previously mentioned, such as training, pharmacist compensation, integrating this new role into pharmacy practice, or setting up physician–pharmacist collaborations for this support.

While this study focuses on connected watches, it would also be relevant to examine other CMDs in different therapeutic fields, particularly in diabetology with diabetes monitoring [31], or in neurology with monitoring of sleep [32], mental health [33], or symptoms of epilepsy [34] or Parkinson’s disease [35]. A larger number of patients, physicians, and pharmacists could be surveyed through a quantitative study.

## 5. Conclusions

This study sought to understand the role of pharmacists in supporting the use of innovative health products outside the pharmaceutical monopoly, specifically in relation to CHOs like connected watches among physicians, patients, and community pharmacists. Interviews were conducted to characterize the use of these watches, patient support, and the expectations of each group regarding pharmacist-led support. This study highlights the crucial role of community pharmacists, whose therapeutic support and patient follow-up skills are essential in patient care. The challenges identified are pharmacist training and compensation for this service, the lack of time for in-depth consultations, and the need for clear and coordinated communication between healthcare providers.

## Figures and Tables

**Table 1:** Characteristics of the study population.

Population	Average Length of Service (Years) [Minimum–Maximum]	Average Length of Interview (Minutes) [Minimum– Maximum]	Place of Working
Rhythmologists	14.5 [2–30]	12.8 [8–17]	University Hospital
Pharmacists Patients	8.2 [1–13] Not Concerned	10.7 [8–12] 12.7 [10–17]	Community Pharmacy Not concerned

**Table 2:** Summary table of the main results of physician interview sessions.

Statement	Number of Physicians in Agreement with the Statement (*N*total = 4)	Proportion of Physicians in Agreement with the Statement
Diagnosis with watches	4	100%
Follow-up with watches	3	75%
The benefits of pharmacist collaboration	3	75%
The pharmacist’s role: Technical support	3	75%
Intuitive use of watches by patients	2	50%
Elderly people have difficulty using watches	1	25%

**Table 3:** Summary table of the main results of patient interview sessions.

Statement	Number of Patients in Agreement with the Statement (*N*total = 10)	Proportion of Patients in Agreement with the Statement
Trust in the dispensing pharmacist	9	90%
Obtaining a diagnosis with watches	8	80%
Challenge: pharmacist training	2	20%
The pharmacist’s role: Technical support	5	50%
Over-control felt with the use of connected watches	3	30%
Have already spoken to the pharmacist about the watch	3	30%

**Table 4:** Summary table of the main results of pharmacist interview sessions.

Statement	Number of Pharmacists in Agreement with the Statement (*N*total = 6)	Proportion of Pharmacists in Agreement with the Statement
CMD allow more regular monitoring	6	100%
Challenge: pharmacist training	6	100%
The benefits of pharmacist support	6	100%
The pharmacist’s role: Conducting interviews	3	50%
Risk associated with use: on control	3	50%
Risk associated with use: independence	2	33%
CMD improve compliance	1	17%

## Data Availability

The original contributions presented in this study are included in the article. Further inquiries can be directed towards the corresponding author.

## Abbreviations

The following abbreviations are used in this manuscript:

CMD	Connected Medical Device
CHO	Connected Health Object
EC	European Conformity
ICT	Information and Communication Technology
ECG	Electro Cardiogram
MD	Medical Device

## Appendix A

### Appendix A.1. Patient Questionnaire

**Questionnaire for patients in the rhythmology department**

- **Patient profile**

Which watch do you use? Which laboratory markets it?

For monitoring which pathologies?

How long have you been using this device?

II. **The connected device**

How did you come up with the idea of acquiring this device?

*Probe: Who advised you?*

Can you tell me about the purchase of your watch?

*Probe: How did you get your watch? Where did you buy it?*

What information were you given at the time of purchase?

*Probe: What was explained to you about the watch when you bought it?*

How often do you use the watch? How much time per day? per week?

*Probe: Do you use your device every day?*

How do you manage the data emitted by your watch?

*Probe: What do you do with the data?*

What has this watch done for you in terms of managing your pathology? What does it do for you on a daily basis?

*Probe: How is your system useful?*

Have you observed any changes in management or medication as a result of using your watch?

Describe an example of a situation in which you found your watch helped you.

Describe an example of a situation in which you felt your watch gave you difficulty or did not help you enough.

What do you think of the financial investment in your watch?

Do you have any concerns about the storage of your personal data?

*Probe: Have you ever wondered how your data is stored/used?*

What role does the watch play in your life, and how do you feel about it?

*Probe: Do you notice any psychological impact from using the watch?*

III. **Support**

How and who else uses the data emitted by your watch?

*Probe: Which healthcare professionals or members of your entourage use your watch?*

How often? How many times a day? A week? Monthly? And for what reasons?

*Probe: How many times a day? Per week? A month? And for what reasons?*

Who would you turn to if you had an emergency message or questions?

*Probe: Who can help you if you have a problem?*

IV. **The pharmacist**

Do you regularly visit the same pharmacy?

*Probe: Do you have a regular pharmacist?*

What is your relationship with your dispensing pharmacist?

*Probe: Do you trust your dispensing pharmacist?*

Have you already discussed your watch with your pharmacist?

*Probe: Have you had any interactions with your dispensing pharmacist regarding the device?*

In what type of situation would you turn to your pharmacist?

How can the pharmacist help you to use your watch?

Does it reassure you to know that your dispensary pharmacist is trained in the use of these watches?

### Appendix A.2. Physician Questionnaire

**Questionnaire for physicians in the rhythmology department**

- **Physician profile**

Seniority?

Most frequently treated pathologies?

II. **The connected device**

What types of connected watches do you use with your patients? Can you cite references? laboratories? examples? On what basis do you prefer one device to another?

How do you use the devices with your patients? How do you advise them to use the device?

What do you use the data emitted by the device for?

*Probe: What do you do with the data?*

What do the devices bring you in terms of care?

*Probe: What aspects of your practice do you find the devices useful for?*

What are your beliefs about the use of these devices?

III. **Support**

How do you support patients and their devices? How often?

*Probe: What roles do you play in supporting patients and their devices?*

How are you approached by patients using this device? How often? How many times a day? Weekly? Monthly?

*Probe*: How often do patients come to you?

IV. **Pharmacist**

Do you work in collaboration with retail pharmacists? How often? How many times a week? Monthly? Yearly?

Do you think a collaboration could be beneficial? If so, why and in what situation?

*Probe: Do you think the dispensing pharmacist could be a local contact for your patients?*

What are your expectations of the dispensing pharmacist?

*Probe: What do you expect from pharmacists?*

What are the points to bear in mind when working together?

If the role of the dispensing pharmacist involved contact between the dispensing pharmacist and the doctor, how should this be done/what tools should be used/what is the preferred method of communication?

### Appendix A.3. Pharmacist Questionnaire

**Questionnaire for pharmacists**

- **Pharmacist profile**

Status?

Seniority?

II. **Connected devices**

What types of devices have you encountered in general? What about cardiology?

What advantages do you see in using these devices for patients?

In the course of your practice, have you come across concrete examples where these devices have improved patient care?

What drawbacks/risks do you perceive in the use of connected devices in cardiology?

III. **The pharmacist’s role in support**

What role do you think pharmacists could play in supporting patients using connected cardiology devices?

*Probe: Could the dispensary pharmacist support patients with a connected device? If so, how?*

In practice, how do you see pharmacists contributing to the monitoring of patients using these devices?

*Probe: Do you have any concrete ideas for implementing follow-up care in pharmacies?*

On which connected cardiology devices could the dispensing pharmacist intervene?

What advice could you give to patients using connected cardiology devices?

*Probe: What connected devices could you advise on?*

If the role of the dispensing pharmacist involved contacting the doctor, how should this be done/what tools should be used/what is the preferred method of communication?

What challenges do pharmacists face in integrating support for these patients into their pharmacy practice?

What are the potential levers for meeting these challenges?

Do you think training is needed for pharmacists to better support the use of these devices?

*Probe: Do pharmacists need training on connected devices?*

What tools or resources would help pharmacists support patients with connected cardiology devices?

*Probe: If you have a difficult question or a sticking point, do you know where you can find the answers/tools to help you?*

## Appendix B

### Appendix B.1. Cross-Sectional Analysis of Physician Interview

**Medical Interviews**		
**Themes**	**Codes**	**Verbatim**
**Device**	**Patient equipment**	“It’s either patients who already have Apple Watches.” “I think it depends on how patients are equipped.” “It depends on the phone the patient has.”
	**Price**	“Choose a model that’s not too expensive.”
	**Quality**	“What we prefer is good quality.”
	**Watch references**	“It’s not bad from Withings^®^ and also we see a bit of Samsung ^®^.” “If they don’t have an Apple Watch^®^ I give them the references for the Whitings^®^ or the Fitbit ^®^.” “I prefer Withings^®^ because it goes with all phones.” “There’s one brand that I use the most, and that my patients buy quite extensively, and that’s Withings^®^.” “I recommend one watch in particular that is compatible with all phone models: Whitings^®^.”
**Patient use**	**Recordings**	“Often it’s they actually try to record and sometimes they can send by e-mail by contacting our office the tracings record.” “These are patients who have palpitations that they are recording at the time.” “We ask them to send us PDF of the ECGs they have recorded by e-mail, explaining their symptoms.” “So the instructions, in quotation marks, are for them to do tracings for me when they’re feeling well, to have a reference of their normal tracings, and to record a tracing when they have a symptom, and then once they’ve done that they send it to me, I tell them they can send it to me by e-mail.”
	**Ease of use**	“Yes, it’s quite easy to use.” “Yes, well, curiously enough, these tools are quite easy for the patient to use and don’t really require much learning.”
	**Patient typology**	“I use it for patients with rare but fairly frequent symptoms.” “When patients come to us complaining of palpitations, sensations of rhythm disturbances that have never been recorded.”
**Use by** **the doctors**	**Diagnosis**	“It gives us a positive diagnosis.” “Either we see that there’s no arrhythmia and there are no abnormalities in that case, well, that’s pretty reassuring, and if not, sometimes it can be dubious, but often we still see that it’s either normal or pathological.” “It allows us to know more precisely what the patient has, to make a diagnosis.” “This will enable us to diagnose and correctly manage the right arrhythmia, because managing atrial fibrillation is not the same as managing tachycardia or other conditions, which are not at all the same treatments.” “Most of the time we use them during the diagnostic phase.”
	**Treatment**	“This can lead directly to a strategy of ablation or drug treatment.” “And if need be initiate a treatment or an intervention behind.”
	**Follow-up**	“Diagnostics! After all, they bought it, so it belongs to them and we can use it to monitor the effectiveness of our treatments.” “It’s for diagnosis at the moment, afterwards, for follow-up, let’s say no more symptoms, he’ll let go of the recordings a little and if there’s a resurgence of symptoms afterwards, they’ll use the device again.” “But it can also be used to evaluate the effectiveness of a treatment.”
	**Patient typology**	“I use it for patients with rare but fairly frequent symptoms.” “When patients come to us complaining of palpitations, sensations of rhythm disturbances that have never been recorded.”
**Added value**	**Qualitative**	“Good quality tracks.” “Tracks are still of fairly good quality.”
	**Efficient diagnostics**	“Diagnostic means for palpitations are pretty poor because it’s either an ECG holter.”
	**Long recording**	“Longer recordings but then it’s over a week or they have to wear one of the holters, which is quite restrictive and fragile.”
	**Ease of use**	“It’s really quite simple to use.”
**Financing**	**Budget**	“We can’t give them too much of a model to equip themselves with, it’s also a matter of their budget.” “But for those who have an iPhone or the means to get a slightly higher-end watch.”
	**Financial brake**	“After all, there is a filter linked to financial investment.” “There are departments that have chosen to invest in the purchase of tools at the hospital, but we have chosen not to do this because it creates additional constraints in terms of equipment, and it also allows us to filter patient motivation to a certain extent.” “Because, on the other hand, it’s a purely personal opinion, but I absolutely don’t want watches to be reimbursed. Patients have to make the investment themselves, otherwise they’ll be left to their own devices.”
**Support from the doctor**	**Solicitation**	“Well, I’d say quite simply, so maybe the other rhythmos will tell you something else, but frankly I don’t think so.” “I don’t get a lot of requests afterwards, because I don’t have many patients with watches, so it’s still filtered, but I’d say like conventional patients.” “In general, we don’t have any questions about the pure use of the watch, that’s more likely to come from the stores.”
	**Recording**	“I ask them to buy the watch, so it’s often the Withings^®^ during a first consultation, for example, and then the instruction is to make tracings as soon as they have symptoms and send them to me by e-mail.” “In fact, we ask them to come and see us again once they have registered symptoms, i.e., it depends a little on the frequency of the symptoms.”
	**Sending tracks**	“Personally, I don’t ever ask for it to be sent to my e-mail address, because otherwise it would mean being available 24 h a day, which is a bit complicated. And what’s more, when you send it to us by e-mail, there’s a medico-legal responsibility.”
	**Meeting**	“That way, at least you’re sure to have tracings at the time of the consultation, because if I see them again in 6 months and they haven’t had anything in 6 months, there’s no point in setting up the appointment in advance.” “But I can’t always reply by e-mail, and if they have a route, we’ll meet again to analyze it together.” “So they come to see us or we can give them a generic mailbox, especially from our telecardiology nurses, but not on personal mail.”
**The pharmacist’s role**	**Complex patients**	“These are patients who require complex care.”
	**No need to**	“I don’t really see what the pharmacist could contribute.” “I’ve never had any feedback from a patient who’s had problems using it.” “I’ve always had the impression that it worked well on its own, spontaneously.” “I’ve never had any feedback from a patient at this level, so I have the impression that it works well.”
	**Technical support**	“An operating aid.” “A handling aid.” “The patient could come to the pharmacy to get a recording with his equipment.” “On a slightly more practical note, to help him properly record his rhythm disorder, palpitations, check that he’s also using it the right way.”
	**Large population**	“It may be possible to extend this to people who are more fragile and have more difficulty understanding. I can see what’s involved in remote monitoring of implantable medical prostheses, and we need more support to reassure people. I think that if we want to extend this kind of device to older people, it could be interesting to have pharmacists involved in this project.” “Reach a wider population to compare with something comparable that is between the doctor and the pharmacist, that is the patient who has just taken their blood pressure at the pharmacy.”
	**Communication**	“He’d have to have more or less secure messaging and things like that. And then the development of networks to enable advanced practices. For example, in our company, we could have privileged contacts, I think paramedics, who could have managed this kind of alert and then redirected patients to doctors according to their profiles, and so on.” “Mail is the easiest way, or via MonSisra^®^, the secure messaging system, but processes need to be put in place, especially if the number of patients increases.”
	**Relationship**	“Yes, I think so, because patients often have a lot of contact with their dispensing pharmacist if they take medication every month or if they go there for other reasons.”
	**Board**	“On an advisory role because it can also have a role if we teach it to validate anomalies.”
	**Training**	“We need to teach the pharmacist how to diagnose atrial fibrillation, for example, which is the most common rhythm disorder.”
	**Limits**	“But I don’t quite see what the pharmacist would get out of it, unless these devices are sold in pharmacies and not reimbursed, in which case why not.” “But only a cardiologist can interpret the trace.”

### Appendix B.2. Cross-Sectional Analysis of Patient Interview

**Patient Interviews**		
**Themes**	**Codes**	**Verbatims**
**Added value**	**Diagnostic utility**	“No matter how much I explained things, as I never had a trace, it was difficult to make a real diagnosis.” “When I showed them to people, they said, “Oh, yes, that’s exactly what you were describing.”” “It argued what I was describing.” “I gave myself this watch, and as soon as I set it, it says “but you’re in atrial fibrillation.” “She’s already discovered it, and now I can keep track of it all.” “I was able to confirm my symptoms and get a more accurate diagnosis”. “And so afterwards I was able to have an operation”. “Because when I went to the doctor for an electrocardiogram, he couldn’t see anything, but you see it’s normal given that I wasn’t feeling my symptoms at the time, otherwise it’s no fun.” “A diagnosis I made a lot a lot of holter of 24, 48 and a week. But there was really no frequency of tachycardia attacks it was really just coming like that.” “The doctor had told me that it helps a lot in the diagnosis and that otherwise I think he would have had a bigger delay in my diagnosis.” “You’re diagnosed faster.” “It’s fast, you don’t have to wait for an appointment”. “It allowed us to be operated on and have an ablation”. “It allowed me to know what I had, because I was told it was stress but I knew it wasn’t.” “It allowed me to undergo surgery and heal, so it brought me health” “Well, I don’t know at the moment, since I’ve got my watch I haven’t had a tachycardia attack yet, so it’s complicated to tell you”. “For the moment I’m waiting to see, it’s too early I think to know that.”
	**Follow-up**	“Now I use it to check that everything is fine and that I’m still in sinus.” “The watch alerts me when I have bradycardia after taking bisoprolol, for example.” “From time to time a little check to see if the rhythm and heart rate are fine.” “To make sure everything goes smoothly.”
**Financing**	**Value for money**	“The watch is quite expensive, at least 150–200 euros”. “I bought it for 450 euros.” “It’s an Apple^®^ Watch and Apple^®^ is expensive.” “But then it’s a cost.” “It’s a great investment in terms of care.” “In terms of the service it provides and the well-being it gives me.” “I think the cost is still right.” “But it’s a necessary investment that’s paying off.” “I think it’s worth it.” “I think it’s a good investment.” “An Apple^®^ Watch is pretty expensive, like everything else from Apple^®^. But I like this brand, I think it’s quality and complete”.
	**Refund**	“I said to myself, ‘It’s not right that this isn’t reimbursed by social security, because we’re saving them money, because every time they go to the doctor for an electrolysis, it costs them money.”
**Therapeutic education**	**Choice of acquisition**	“I asked around and bought a Withings^®^ watch”. “Yes, I looked it up on the internet. No one recommended it to me.” “Afterwards, I did a lot of research on the Internet myself, so I already knew a bit about it.” “My husband bought it in a store, I think it was a birthday present.” “A relative advised me to do this.” “So I came up with the idea of buying my watch after having difficulty getting a precise diagnosis from the doctors I saw. And so once in consultation my doctor told me about it and suggested the idea.” “The doctor told me it was great to buy this watch.” “At the time, our rhythmologist advised us to do so.” “It was our rhythmologist who advised us to invest in these watches.” “It was my cardiologiste who advised me to take the watch, and he gave me the brand name too.” “I was advised to buy a watch in consultation to see what I really had.”
	**Information**	“In the store, I met a gentleman who knew it very well, since his mother owned one, and it was he who guided me.” “So the salesman explained to me in detail how the watch works, because there are so many functions on it.” “Yes, a little. I asked the salesman and he explained everything to me.” “I looked on the internet and then I saw that it was the one that could best match what I had.” “I didn’t feel well and it was a bit like what had happened 16 years ago. So I said to myself, without thinking about it, I’m going to buy myself a watch to follow my heart.” “So when I got home I did some research online and opted for the Fitbit because it seemed to fit my needs.” “I found out about the feature directly on the Internet.” “No, there was the notice in the package.” “It’s the doctor, but it’s quite easy to use. The most complicated part was installing the software, after that it’s a piece of cake.” “Our doctor explained to us how to use it.”
**Patient use**	**Frequency**	“Often.” “I do an ECG every week.” “I use it every day, or at least I wear it every day.” “I carried it with me all the time and when I had problems I knew I could use it and do an ECG.” “Before the operation it was once a day and a little less, rarely every day.” “I used to use it very often, every day. But now I look at it from time to time, I think twice a week.” “My watch is always with me and I use it when I don’t feel well. Not every day, but several times a week”. “It’s once or twice a day, it’s not very frequent.” “Now a little less than at the beginning but I continue to wear it every day, I do less tracing maybe 1 per week because I am healed.” “Every day. For a lot of things, whether it’s the sleep app, sms, health and for recordings.”
	**Autonomy**	“I’m totally autonomous.” “No one but me.” “No, no, all alone.” “I text the ECG to my sister and if it’s abnormal my sister tells me to go to the doctor.” “My wife sometimes, as we have the same problem and both watches, we do together.” “And it was my husband who installed everything on our phones and linked it to the watch.” “Yes, my husband helped me at the beginning to set everything up.” “Yes, my husband helped me. He’s the one who told me about this job and how it worked.” “My friend had given me a briefing.”
	**Difficulties**	“If it’s a bit anarchic, sometimes the recording is interfered with. No matter how many times I redo it, redo it, redo it, it doesn’t happen.” “It’s just at the beginning of the application installation.” “If it hadn’t been for my husband in the beginning, I think I would have needed someone’s help, at least in the early stages.” “I didn’t need an explanation from the pharmacist because my friend was there, but otherwise I could have asked for help, yes.” “No, it’s fine, my son explained everything to me and I’m very comfortable technology, I’m curious.” “Not really.” “Because it’s easy enough to make an ECG, but it’s hard enough to find it in the Health app, and to interpret it too.” “Not at all, it’s very easy.” “No, never.”
**Psychological impact**	**Positive**	“It was rather reassuring.” “The watch really helped me tell myself that it wasn’t just psychological and diagnostic.” “When I look at her it reassures me” “It gives me peace of mind that the operation is still reliable.” “I use the ECG sometimes when I have fears or psychoses, it happens to me… And then it reassures me to see that the ECG is totally normal so I go on with my day.” “But then, on the one hand, it’s reassuring to have it, because if I have a tachycardia attack in the street, I can do an ECG and since I’m already being monitored, we can find out what happened.” “None, we do it when we have a little worry if we feel it throbbing a little bit.” “And for me, the watch is a comfort, because I know that if I have a problem, I can record it and the doctors will be able to treat me.” “It’s true that I have a slight tendency to do ECGs at the slightest symptom when I’m not having an attack.”
	**Negative**	“I tend to want to go nowhere without it.” “I’m a bit of a slave to the watch, but I’m interested.” “But frankly, I used to be a bit of a hypochondriac.” “Ah yes, what isn’t always very positive can have a real perverse effect too.”
**Physician support**	**Instructions**	“Really it was very intense or very violent or clearly the watch is telling me something abnormal because sometimes it says so, in which case I email it directly to the rhythmologist” “After it was recorded in the Apple^®^ health application, I would either send the trace to be exported as a PDF or I would send the trace by e-mail to the doctor or to my sister, who is a doctor.” “If there’s nothing, I don’t move, because the device tells you if you have an arrhythmia, etc. If the device is there, I send it to the rhythmologist with a note by e-mail and then make an appointment.” “In general, I do nothing because it’s business as usual, but if it seems abnormal, I send it to the doctor, who then tells me what to do.” “In fact, they’re recorded on my cell phone in an application whose name I’ve eaten… But it’s the application that’s linked to the watch. And I send it to the doctor when I have a tachycardia attack that’s triggered. For the moment, it hasn’t happened, but twice I’ve sent the ECGs I did during an acceleration to my doctor, but they didn’t see anything on the ECGs, so I’m instructed to continue recording and sending them to get a diagnosis”. “I’m instructed to do tracings at the slightest symptom, and if it’s abnormal I send it to the department secretary. Then the secretary emails me the doctor’s feedback. For the moment I haven’t had a new consultation, I’ve sent a trace but nothing conclusive apparently, so I’m continuing.” “I just look at them, at first I showed them to the doctor and then they operated on me. But now it’s just for my own information, I keep tracking my condition.”
	**Follow-up**	“In there is a problem I send it to the cardiologist or rhythmologist directly” “If I have a problem, I contact my cardiologist directly, or my general practitioner if the cardiologist doesn’t answer. And if it’s very urgent, I call the emergency services.” “I can also send it to the doctor to tell me what to do”. “We send her an e-mail and she replies very quickly that she knows how to make herself available.”
**The role of the dispensing pharmacist**	**Patient type**	“For the operation, someone who wouldn’t actually arrive” “Yes, maybe for people who have a bit more difficulty.” “Yes, it could be a plus, especially for people less at ease with watches.”
	**Proximity/trust**	“Absolutely, I’ve maintained a relationship of trust with them for 25 years”. “Yes I showed it to the team and told them my story, we talk a lot.” “Yes, very much.” “Yes of course” “By definition, I trust pharmacists.” “Yes, totally.” “I don’t know him very well, but he’s okay medically, yes”. “I talked about it with my friend, yes. He knows a bit about all this and then the younger members of the team too, they’re well connected themselves so it interested them too.” “Yes, to tell the story of what happened when I came back from hospital”. “Yes, a little with the girls from the pharmacy, we chat sometimes when they have time. I told them a bit about my hospitalization, so of course I mentioned the watch”. “It’s difficult to establish a real relationship in the end. I’m welcomed and taken care of, but to say that we’ve been able to establish a relationship of trust, I’d say no.” “No, I didn’t know I could talk to him about it, so I didn’t”. “No, I haven’t been since I got my watch”.
	**Training**	“It’s in everyone’s interest to wear this watch and for pharmacists and doctors to be trained in the use of these watches to help and reassure us”. “That’s for sure. It’s even better because we don’t know what can happen, so it’s better for doctors, pharmacists and even nurses to be trained”. “I think it needs to do training tailored to that anyway”.
	**Follow-up**	“Yes, of course, I’m not sure how, but it could be great to do little check-ups with them. My mother received a paper from the health insurance company to do a check-up with her pharmacist, and I think we could do that too.” “For reading electrocardiograms, I don’t think so”.
	**User help**	“Yes, it’s true that it could be a plus to be able to go to the pharmacy and get help, like when you go to take your blood pressure. Being able to get help and a little support sometimes feels good”. “If he has time he could help to install the software on the laptop and do a first test with the patient”. “I’ve got a health problem and I can find answers on the Internet, because that’s what today’s society is all about: we tend to go straight to the Internet to explain our doubts about something. I’d go and see my pharmacist and ask her what she thinks”. “Maybe when I first bought it I could have asked him directly instead of spending time on the internet. With the internet, you don’t always know whether you’re reading things that are true or false”. “I’d say that, like nurses, we’re capable of reading an ECG and detecting emergencies so we can redirect. It’s true that my sister is a doctor, so I send it to her and she tells me whether or not I’m going to the emergency room, but for someone else, the pharmacist can play this role”. “I don’t know if he can sell them in his pharmacy. But he can help us if we’re having trouble. If it hadn’t been for my husband in the beginning, I think I’d have needed someone’s help, at least in the beginning. After that, it’s a breeze.” “Yes, certainly, to explain how it works. I didn’t need an explanation from the pharmacist because my friend was there, but otherwise I could have asked for help, yes.”
	**Limits**	“But there’s nothing in it for him, let’s say.” “The pharmacist is not involved because it doesn’t require consumables or anything so…”

### Appendix B.3. Cross-Sectional Analysis of Pharmacist Interview

**Pharmacist Interviews**		
Themes	Codes	Verbatims
Connected medical device	In general at the pharmacy	“Blood glucose test.” “TENS” “Tensiometers”
	In cardiology at the pharmacy	“Tensiometer” “Oximeter” “Watches” “Ring”
	Benefits	“Follow-up, control, autonomy” “At the pharmacy, it allows us to adapt our advice and provide guidance” “Enhance our status by explaining the steps to follow” “Improved compliance and follow-up” “More regular follow-up” “Continuous care for the patient”
	Disadvantages	“To consult less” “One of the main risks, in my opinion, is the proliferation of high-tech DM and mainly connected devices, and their sale outside pharmacies, for example blood pressure monitors at supermarket.” “The place of purchase outside the medical circuit” “Generated stress and over-control.” “Hypochondriac.” “Dysfunction.” “Individual follow-up” “Spending too much time checking on the connected medical device.”
Compendial practice	Past experiences	“Self-measurement by patients, yes I have examples where there has been a reduction in hypertensive treatments following low blood pressure readings.” “Midwives monitor blood sugar levels during pregnancy for certain patients via blood sugar meters directly connected to an app.” “The majority of my patients have their blood pressure monitored at home and this improves their management.” “Diabetes yes, patients with the devices have much more regular follow-ups which enable them to adapt to variations in their live levels.”
	Installation	“At the counter you can ask for the values or if the device is working”. “No, we’d have to think about it, but I don’t think we have enough time, and the product would have to be sold in pharmacies or there would have to be some kind of remuneration to motivate people.” “By appointment or at the counter.” “Like therapeutic interviews.” “It has to fit in with the pharmacy cycle and perhaps be remunerated to encourage more.”
Support pharmacist	Role	“Use” “The link between values and disease” “On what to do” “Reassuring the patient” “Accompaniment but something quite practical as a frontline healthcare professional.” “Practical help”
	Brakes	“If the devices are used on patients with cardiac pathologies that are a little specific or too specialized, we won’t be trained for it, so I’m afraid the patient will end up knowing more and we’ll be useless” “Lack of time, but also lack of knowledge”. “Lack of openness and training too” “Lack of medical knowledge” “Lack of knowledge about connected medical device”
	Levers	“Training, And for the lack of time maybe make appointments like for interviews” “Slots and appointments, remuneration, training”
	Resources	“Laboratory training, and reminders of associated pathologies” “Technical data sheets or tutorials for devices”
Working with doctors	Communication	“Secure messaging or by e-mail” “It would be great to have a number accessible by pharmacists or a fax like they already do in Canada to have privileged access.” “Mail or secure messaging and by phone if it’s really an emergency”. “In case of emergency, the telephone”
